# Can PD-L1 expression be predicted by contrast-enhanced CT in patients with gastric adenocarcinoma? a preliminary retrospective study

**DOI:** 10.1007/s00261-022-03709-9

**Published:** 2022-10-21

**Authors:** Xiaolong Gu, Xianbo Yu, Gaofeng Shi, Yang Li, Li Yang

**Affiliations:** 1 Department of Radiology, The Fourth Hospital of Hebei Medical University, Shijiazhuang, Hebei 050011 People’s Republic of China; 2 CT Collaboration, Siemens Healthineers Ltd., Beijing, People’s Republic of China

**Keywords:** Radiomics, PD-L1, Gastric adenocarcinoma, Computed tomography

## Abstract

**Background:**

This study aimed to construct a computed tomography (CT) radiomics model to predict programmed cell death-ligand 1 (PD-L1) expression in gastric adenocarcinoma patients using radiomics features.

**Methods:**

A total of 169 patients with gastric adenocarcinoma were studied retrospectively and randomly divided into training and testing datasets. The clinical data of the patients were recorded. Radiomics features were extracted to construct a radiomics model. The random forest-based Boruta algorithm was used to screen the features of the training dataset. A receiver operating characteristic (ROC) curve was used to evaluate the predictive performance of the model.

**Results:**

Four radiomics features were selected to construct a radiomics model. The radiomics signature showed good efficacy in predicting PD-L1 expression, with an area under the receiver operating characteristic curve (AUC) of 0.786 (*p* < 0.001), a sensitivity of 0.681, and a specificity of 0.826. The radiomics model achieved the greatest areas under the curve (AUCs) in the training dataset (AUC = 0.786) and testing dataset (AUC = 0.774). The calibration curves of the radiomics model showed great calibration performances outcomes in the training dataset and testing dataset. The net clinical benefit for the radiomics model was high.

**Conclusion:**

CT radiomics has important value in predicting the expression of PD-L1 in patients with gastric adenocarcinoma.

## Introduction

One of the leading causes of cancer deaths worldwide is gastric cancer [1]. Gastric adenocarcinoma is the most common pathological type, and immunotherapy has shown application prospects. For gastric adenocarcinoma, tumor heterogeneity is a challenge in diagnosis and treatment, and tumor gene sequencing provides a potentially valuable resource [2]. Immunotherapy and targeted therapy are effective methods for the treatment of gastric adenocarcinoma. Biomarkers such as programmed cell death-ligand 1 (PD-L1), human epidermal growth factor receptor 2 (HER2), and microsatellite instability (MSI) gradually promote systemic therapy [1]. Clinical studies have shown that PD-L1 combined with chemotherapy can benefit patients with gastric adenocarcinoma and has acceptable safety [3, 4]. PD-L1 expression has been shown to be a tumor marker that can predict the response to targeted therapy in patients with gastric adenocarcinoma [5].

Immunohistochemistry is the gold standard to determine the expression level of PD-L1 in pathological specimens, but it cannot be implemented in patients in whom it is difficult to obtain samples, and the diagnosis process takes a long time. If the samples are taken under an endoscope, the diagnostic accuracy is affected due to the heterogeneity of the tumor. As a noninvasive and rapid diagnostic method, computed tomography (CT) is widely used in clinical practice. Radiomics data can be used to develop models to provide evidence for cancer immunotherapy [6, 7]. Previous studies have shown that quantitative radiomics features based on CT can be used to predict the expression of PD-L1 in small cell lung cancer and provide information for clinical immunotherapy [8, 9]. Other studies have shown that radiomics models can predict the prognosis of metastatic urothelial cancer patients receiving immunotherapy [10]. Thus, the relationship between radiomics features and PD-L1 expression in gastric adenocarcinoma is worth studying.

Radiomics is increasingly being used to predict immune markers. The aim of this study was to establish a radiomics model based on CT in gastric adenocarcinoma to predict PD-L1 expression.

## Methods

### Study design and study population

This retrospective study was approved by the ethics committee (protocol number 2022KY235); the requirement for written informed consent was waived owing to the use of deidentified retrospective data. Patients were enrolled from January 2020 to November 2021. The inclusion criteria were as follows: (1) patients underwent surgical resection with no distant metastasis; (2) patients had confirmed gastric cancer by histology and underwent PD-L1 testing; and (3) patients underwent pretreatment contrast-enhanced abdominal CT scanning. The exclusion criteria were (1) previous anticancer therapy, (2) incomplete clinical data, and (3) CT images that were incomplete or of poor quality.

### The clinical evaluation index

One author analyzed the clinical features of the patients. The following clinical features were recorded: (1) sex, (2) age, (3) smoking history, (4) drinking history, (5) Eastern Cooperative Oncology Group (ECOG) score, (6) location of gastric adenocarcinoma, (7) tumor (T) stage, (8) node (N) stage, and (9) degree of differentiation.

### Immunohistochemistry and evaluation of PD-L1 expression

All postoperative tumor specimens were subjected to immunohistochemistry (IHC) according to a standard procedure. The samples were processed as follows. The use of formalin-fixed, paraffin-embedded tissues has been validated. Specimens were sectioned to a thickness of 4 mm, fixed in formalin, dehydrated and cleared in a series of alcohols and xylene, followed by melted paraffin infiltration. The samples were fixed for 12 to 72 h in 10% neutral buffered formalin. A three-in-one procedure of deparaffinization, rehydration, and target retrieval was performed. Sections were stained with PD-L1 IHC 22C3 pharmDx (Agilent, USA) on a Dako immunohistochemical staining instrument. When membrane staining was detected in tumor cells, PD-L1 expression was considered positive. The expression of PD-L1 was evaluated by the combined positive score (CPS). The expression of PD-L1 was positive when the CPS score was greater than 1.

### CT acquisition technique and image segmentation

All patients underwent pretreatment contrast-enhanced diagnostic abdominal CT. CT plain scan and arterial phase and venous phase enhanced scans were performed on all patients. All CT examinations were performed using one of three multidetector CT scanners: a 256-detector CT scanner (Revolution CT, GE Medical systems) and two 128-detector CT scanners (SOMATOM Definition Flash, Siemens Healthineers and Brilliance iCT, Philips Healthcare). The scan parameters were as follows: tube voltage 120 kV and tube current using automatic milliampere second technology. The scan ranged from the dome of the diaphragm to the pubic symphysis. For enhanced scanning, nonionic contrast medium (iodine content of 300 mg/ml), an injection flow rate of 3.0 ml/s, and a total contrast medium of 2 mL/kg body weight were used. The arterial phase and venous phase were scanned at 25 s and 70 s after injection. The raw data were transferred to the postprocessing workstation (syngoMMWP, VE36A) to generate images in the venous phase. The reconstruction thickness was 1.0 mm.

**Fig. 1:**
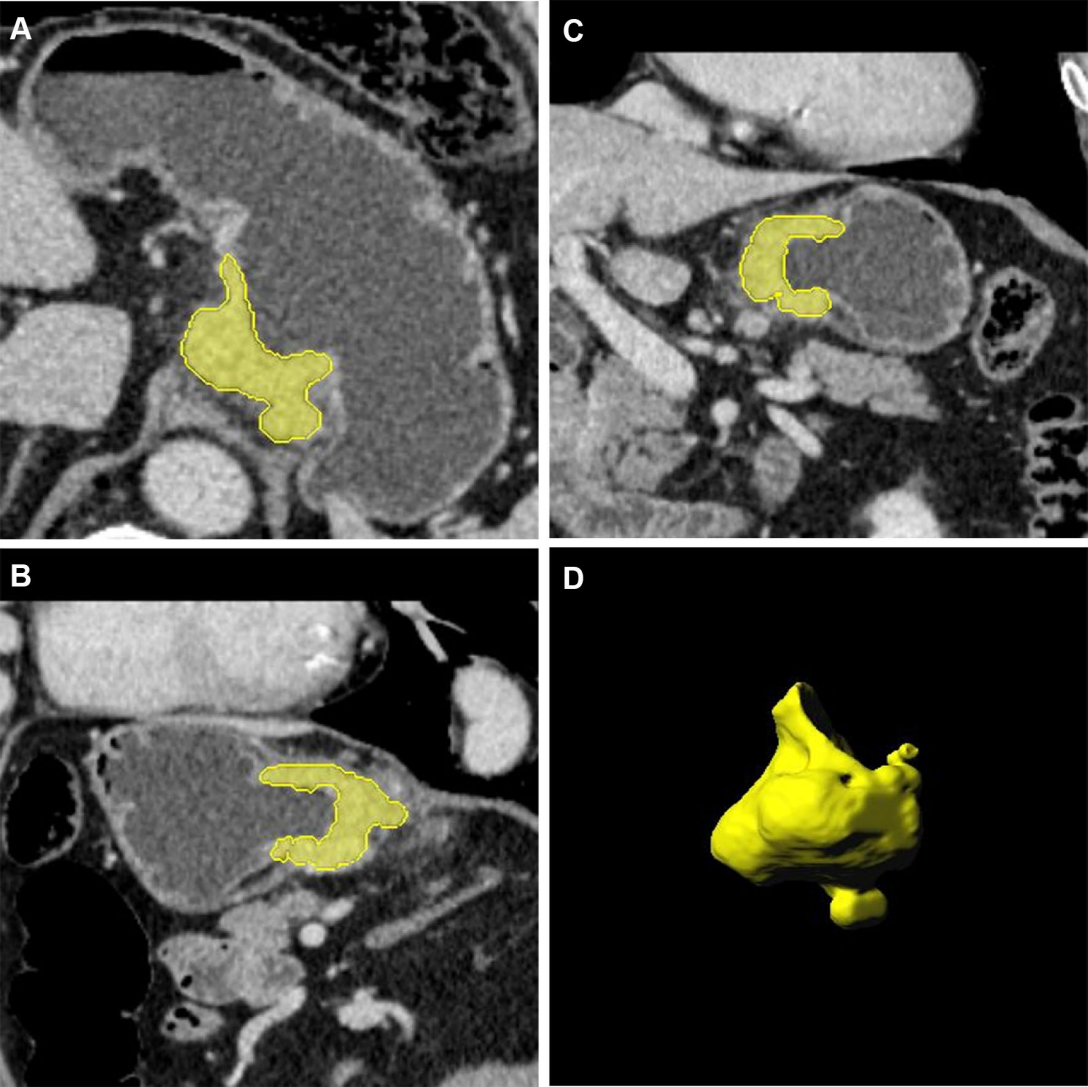
**a**–**d**. Region of interest and the segmented images. **a** Axial position. **b** Sagittal position. **c** Coronal position. **d** Segmented image

For gastric adenocarcinoma tumors, the venous phase images of a slice thickness of 1 mm were semiautomatically segmented by software (Radiomics, Frontier, Siemens Healthineers, Forchheim, Germany). A radiologist (GXL with 7 years of abdominal radiology experience) delineated the ROIs. ROIs were drawn from each of the three positions (axial, sagittal, and coronal) in turn. The tumor boundary of the delineation slices was validated by another radiologist (YL with 19 years of experience in abdominal radiology). Consensus was reached through negotiation to resolve differences. The CT image of the region of interest and the segmented 3D image were obtained. Figure 1 shows an example.

### Radiomics feature selection and model development

The training dataset and testing dataset were randomly generated in a 7:3 ratio. The software (Radiomics, Frontier, Siemens Healthineers, Forchheim, Germany v1.2.5), which was a research platform, was used to extract all features and parameters as follows. The following radiomic feature groups were selected: gray level dependence matrix (GLDM) features, gray level co-occurrence matrix (GLCM) features, shape features, first-order features, gray level run length matrix (GLRLM) features, gray level size zone matrix (GLSZM) features, and neighboring gray tone difference matrix (NGTDM) features (Table 1). For filtering, the LoG sigma values (mm) were 0.5, 1.5, 2.5, 3.5, 3.5, and 4.5 (with 5 sigma levels and 1 level of wavelet decomposition yielding 8 derived images and images derived using square, square root, logarithm and exponential filters). The coiflet wavelet was selected. Resampling was not selected due to the consistent layer thickness. The characteristic data were not normalized because doing so could lead to the loss of some features. The bin width when making a histogram for discretization of the image gray levels was 25. To verify the stability of the features, radiologists redrew the ROIs for 50 random cases one month after the first delineation was completed. The intragroup correlation coefficients of the features were calculated. Any feature with ICC < 0.8 was excluded. A total of 434 features remained. The random forest-based Boruta algorithm was used to screen the features of the training set. The cross-verification method was used to select the value. The radiomics model was constructed based on the screened radiomics features. The testing dataset was used to test the performance of the model.

**Table 1:** Summary of the radiomics features

Group of features	Image phase
Gray level dependence matrix	Venous phase
Gray level co-occurrence matrix	Venous phase
Shape	Venous phase
First order	Venous phase
Gray level run length matrix	Venous phase
Gray level size zone matrix	Venous phase
Neighboring gray tone difference matrix	Venous phase

### Statistical analysis

All statistical analyses were performed using R version 4.0.5. Averages and standard deviations, as well as medians and ranges, are used to present the data. The patient characteristics were compared by the Mann‒Whitney U test, χ2 test, and Student’s *t* test. The random forest-based Boruta algorithm was used to screen the features. In random forest, multiple decision trees are generated and then the final results are decided [11]. The receiver operating characteristic (ROC) curve was used to determine the optimal critical value, sensitivity, and specificity of the model. *P* < 0.05 was considered statistically significant. Calibration curve and decision curve analyses were used to assess clinical utility.

## Results

### Patient characteristics

A total of 169 patients with GC who underwent pretreatment contrast-enhanced abdominal CT were included in this retrospective study. The median age was 62 years (range, 55–69). Sixty-five of the 169 GC patients in our study were male, and 104 were female. The expression of PD-L1 was negative in 75 patients and positive in 94 patients. The full results are reported in Table 2. Computer-generated random numbers were applied to assign 118 patients to the training dataset and 51 patients to the testing dataset. In the training dataset, 70 people were positive for PD-L1 expression, and 48 people were negative. In the testing dataset, 24 people were positive for PD-L1 expression, and 27 people were negative. There was no statistically significant difference between the two datasets in PD-L1 expression (*p* > 0.05) (Table 3).

**Table 2:** Clinical Data

Variable	Classification item	Total (*n* = 169)
Sex, *n* (%)	Male	104 (61.5)
	Female	65 (38.5)
Smoking, *n* (%)	No	103 (60.9)
	Yes	66 (39.1)
Drinking, *n* (%)	No	124 (73.4)
	Yes	45 (26.6)
ECOG, *n* (%)	0 points	141 (83.4)
	1–2 points	28 (16.6)
T stage, *n* (%)	Stage 1	8 (4.7)
	Stage 2	9 (5.3)
	Stage 3	5 (3.0)
	Stage 4	147 (87.0)
N stage, *n* (%)	Stage 0	33 (19.5)
	Stage 1	57 (33.7)
	Stage 2	37 (21.9)
	Stage 3	42 (24.9)
Differentiation, *n* (%)	Poorly differentiated	147 (87.0)
	Moderately differentiated	22 (13.0)
Age, median [IQR]		62 [55.0,69.0]
PD-L1 expression	Negative	75 (44.4)
	Positive	94 (55.6)

**Table 3:** Training and testing datasets

	Training	Testing	*p* overall
	*N* = 118	*N* = 51	
PD-L1 expression			0.192
Negative	48 (40.7%)	27 (52.9%)	
Positive	70 (59.3%)	24 (47.1%)	
Sex			0.156
Male	68 (57.6%)	36 (70.6%)	
Female	50 (42.4%)	15 (29.4%)	
Age	62.5 [54.2;69.0]	59.0 [55.0;67.5]	0.297
Drinking			0.976
No	86 (72.9%)	38 (74.5%)	
Yes	32 (27.1%)	13 (25.5%)	
Smoking			0.115
No	77 (65.3%)	26 (51.0%)	
Yes	41 (34.7%)	25 (49.0%)	
ECOG score			0.636
0 point	100 (84.7%)	41 (80.4%)	
1–2 point	18 (15.3%)	10 (19.6%)	
Location			0.981
Cardia	38 (32.2%)	15 (30.0%)	
Fundus	7 (5.93%)	4 (8.00%)	
Body	36 (30.5%)	15 (30.0%)	
Horn	2 (1.69%)	1 (2.00%)	
Antrum	35 (29.7%)	15 (30.0%)	
T stage			0.960
Stage 1	6 (5.08%)	2 (3.92%)	
Stage 2	7 (5.93%)	2 (3.92%)	
Stage 3	2 (1.69%)	0 (0.00%)	
Stage 4	103 (87.3%)	47 (92.2%)	
N stage			0.078
Stage 0	26 (22.0%)	7 (13.7%)	
Stage 1	36 (30.5%)	21 (41.2%)	
Stage 2	22 (18.6%)	15 (29.4%)	
Stage 3	34 (28.8%)	8 (15.7%)	
Differentiation			0.668
Poorly differentiated	104 (88.1%)	43 (84.3%)	
Moderately differentiated	14 (11.9%)	8 (15.7%)	

### Associations between clinical factors and PD-L1 expression

Table 4 shows the relationship between clinical features and PD-L1 expression in the training and testing datasets. None of the clinical features showed apparent differences between the positive and negative PD-L1 groups in the training and testing datasets.

**Table 4:** Relationship between clinical features and PD-L1 expression

	Negative	Positive	*p* overall
	*N* = 75	*N* = 94	
Sex			0.599
Male	44 (58.7%)	60 (63.8%)	
Female	31 (41.3%)	34 (36.2%)	
Age	59.0 [50.0;68.0]	62.5 [56.2;69.0]	0.076
Drinking			0.592
No	53 (70.7%)	71 (75.5%)	
Yes	22 (29.3%)	23 (24.5%)	
Smoking			0.483
No	43 (57.3%)	60 (63.8%)	
Yes	32 (42.7%)	34 (36.2%)	
ECOG score			0.388
0 point	60 (80.0%)	81 (86.2%)	
1–2 point	15 (20.0%)	13 (13.8%)	
Location			0.166
Cardia	22 (29.3%)	31 (33.3%)	
Fundus	5 (6.67%)	6 (6.45%)	
Body	29 (38.7%)	22 (23.7%)	
Horn	2 (2.67%)	1 (1.08%)	
Antrum	17 (22.7%)	33 (35.5%)	
T stage			0.385
Stage 1	2 (2.67%)	6 (6.38%)	
Stage 2	3 (4.00%)	6 (6.38%)	
Stage 3	0 (0.00%)	2 (2.13%)	
Stage 4	70 (93.3%)	80 (85.1%)	
N stage			0.551
Stage 0	12 (16.0%)	21 (22.3%)	
Stage 1	24 (32.0%)	33 (35.1%)	
Stage 2	17 (22.7%)	20 (21.3%)	
Stage 3	22 (29.3%)	20 (21.3%)	
Differentiation			0.050
Poorly differentiated	70 (93.3%)	77 (81.9%)	
Moderately differentiated	5 (6.67%)	17 (18.1%)	

### Construction of the radiomics signature

**Fig. 2:**
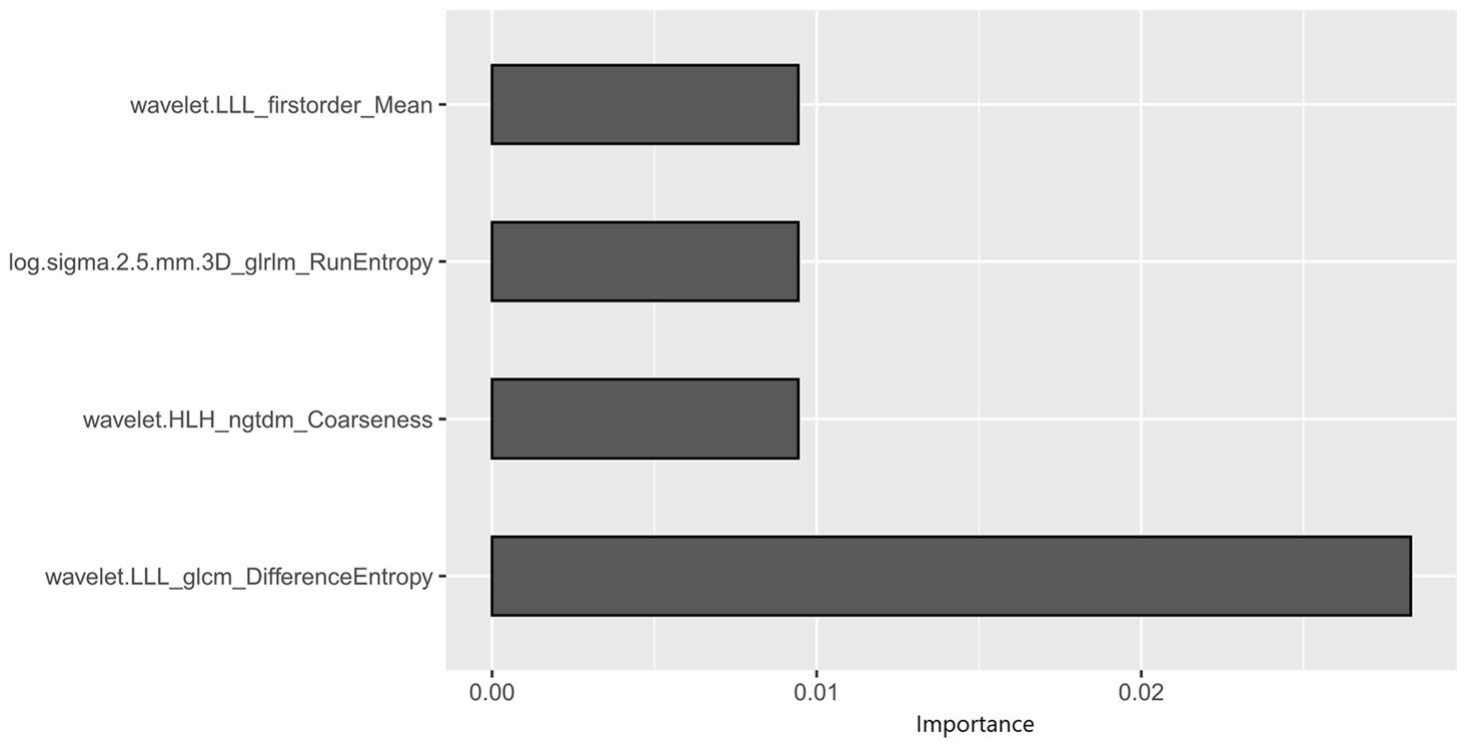
Weights of the four radiomics features in the radiomics model

The radiomics analysis identified 1226 features, all of which were extracted from the segmented pretreatment CT images of 118 patients with GC. A total of 792 features were excluded because they had an ICC greater than 0.8. A total of 434 features were stable. Finally, four features were screened out, including one first-order feature, one GLRLM feature, one NGTDM feature and one GLCM feature. The importance of the 4 radiomics features is shown in Fig. 2. The ‘wavelet. LLL_glcm_DifferenceEntropy’ feature had the largest mean importance.

### Development and testing of the predictive model

**Fig. 3:**
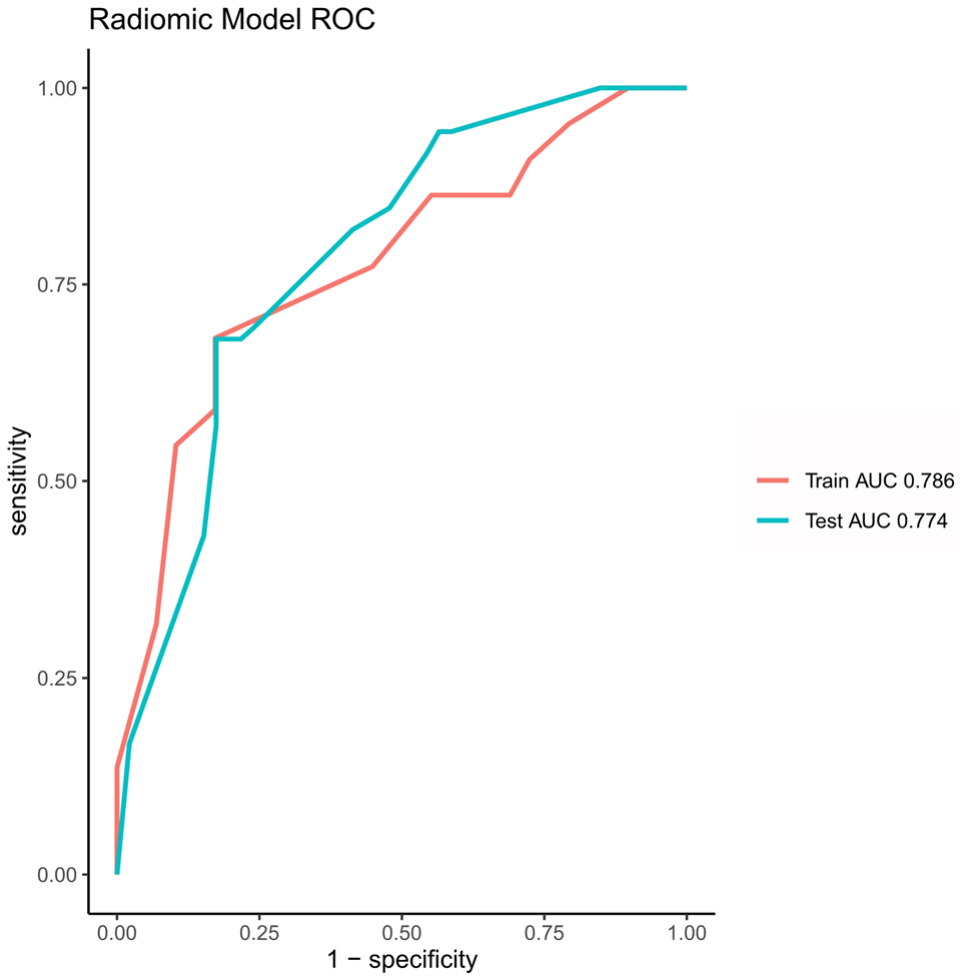
ROC curves of the training dataset (red) and testing dataset (blue)

A radiomics predictive model was constructed based on only four radiomic features since clinical features were not significantly different (Table 4). PD-L1 expression prediction using the above radiomics signature showed a favorable assessment efficacy, with an AUC of 0.786 (*p* < 0.001), a sensitivity of 0.681, and a specificity of 0.826. The radiomics model achieved the greatest AUC in the training group (AUC = 0.786) and validation group (AUC = 0.774) (Fig. 3). Table 5 shows the specificity and sensitivity of the model.

**Table 5:** Performance of the radiomics model for predicting PD-L1 expression

	AUC (95%CI)	Sensitivity	Specificity
Training dataset	0.786 (0.700–0.872)	0.681	0.826
Testing dataset	0.774 (0.640–0.907)	0.591	0.828

**Fig. 4:**
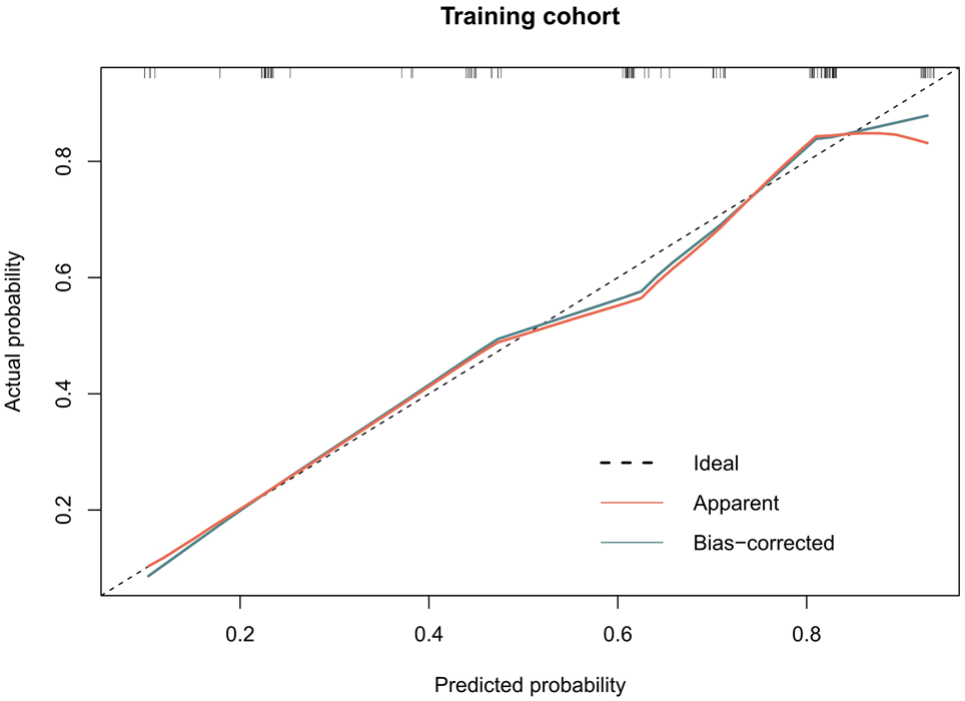
Calibration curve for the radiomics model in the training dataset. The horizontal axis represents the predicted probability of the radiomics feature model, and the vertical axis represents the actual probability. The predictions and observations were in good agreement

**Fig. 5:**
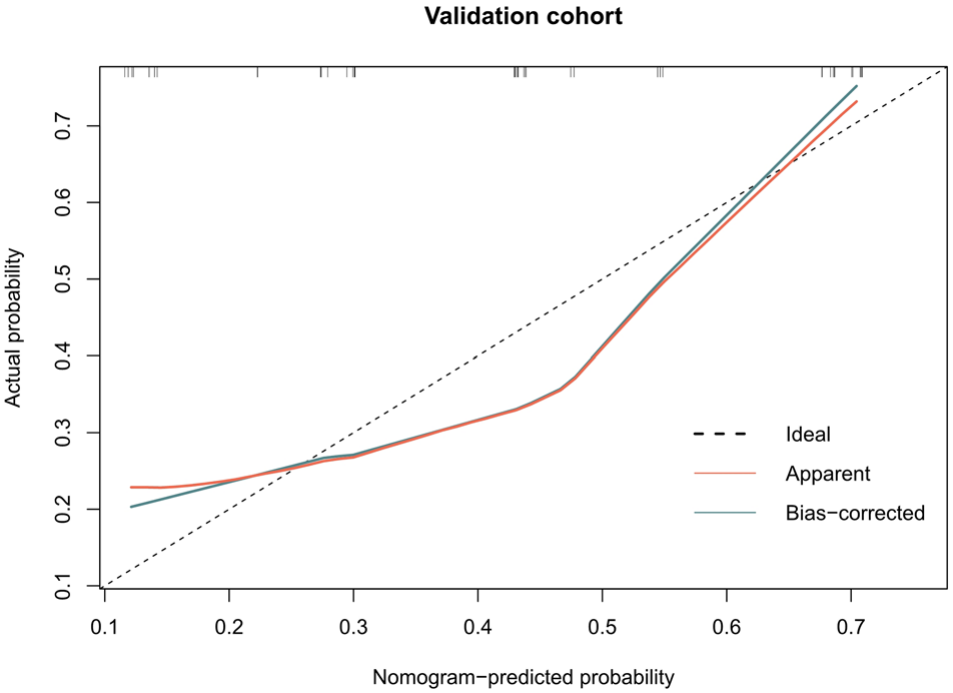
Calibration curve for the radiomics model in the testing dataset. The horizontal axis represents the predicted probability of the radiomics feature model, and the vertical axis represents the actual probability. The predictions and observations were in good agreement

**Fig. 6:**
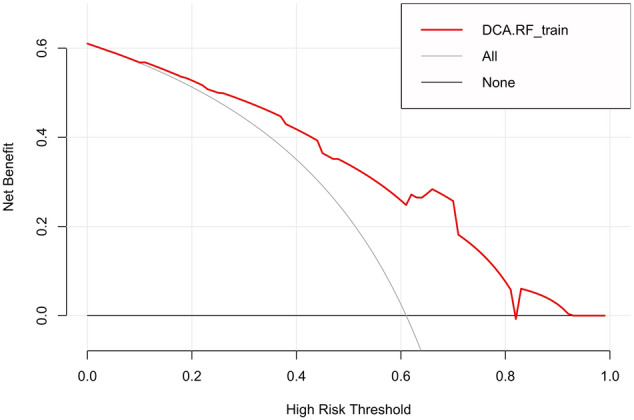
Decision curve for the radiomics model in the training dataset. The x- and y-axes of the curve represent the threshold probability and the net benefit, respectively

**Fig. 7:**
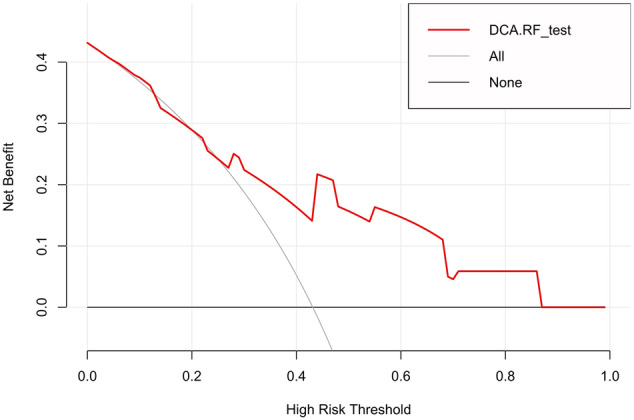
Decision curve for the radiomics model in the testing dataset. The x- and y-axes of the curve represent the threshold probability and the net benefit, respectively

The calibration curves of the radiomics model showed great calibration performance outcomes in the training dataset (Fig. 4) and testing datatset (Fig. 5). The predictions and observations were in good agreement. As shown in Fig. 6 and Fig. 7, the net clinical benefit for the radiomics model was high. This indicates that it is beneficial to use radiomics features to predict PD-L1.

## Discussion

The application value of immunotherapy in the treatment of gastric adenocarcinoma is continuously being revealed. CT radiomics is a noninvasive diagnostic method that can be used to predict PD-L1 expression. Our study shows that radiomics features can be used to construct a model for predicting tumor PD-L1 expression.

Radiomics features with predictive value can be used to build a radiomics model with a good prediction effect. The ‘wavelet. LLL_glcm_DifferenceEntropy’ feature had the largest mean importance. This feature indicates that a process of variable-speed entropy increases has occurred, reflecting the degree of tumor immunosuppression. From the perspective of entropy, life depends on the negative entropy provided by the external environment to maintain the degree of order. Tumors develop from mutated cells, accumulate mutation effects, and have the ability to escape the regulation of the immune system. The tumor absorbs negative entropy from the external environment to keep its own entropy low and keep itself active [12]. The PD-L1 expression level was thus revealed.

A dramatic development is occurring in the field of radiomics for gastric cancers. For example, radiomics has been used to predict T stage [13], N stage [14, 15], M stage [16, 17], neoadjuvant chemotherapy [18, 19], Lauren classification [20], and histological grade [21]. Studies showed that CT radiomics is an important preoperative predictor and provides prognostic information for pathological staging markers [22]. Other studies showed that CT radiomics can be used to distinguish whether EGFR2 is positive [23]. This study showed that the radiomics model is an important preoperative predictor for the expression of PD-L1. Compared with other similar studies, we focused on the study of PD-L1 other than CD8 + TILs, which was also one of the directions of further research [24]. CT radiomics is subjective in the standardization of tumor segmentation methods. The image acquisition parameters may also affect the standardization of radiomics data. Therefore, we used semiautomatic manual adjustment and uniform CT scanning parameters to avoid design bias in the real world.

The selection of the CPS score in PD-L1 research is of significance to discuss. We considered the pathological and clinical significance. For pathology, PD-L1 negativity and positivity were distinguished by a CPS score of 1. The KEYNOTE59 and KEYNOTE61 studies [25] have shown that it is important to select a CPS score greater than 1, and these patients can benefit from immunotherapy. This could be further studied in future research.

The details of tumor segmentation and the selection of radiomics features are important to maintain the prediction level of the model. Three-dimensional sketches are used to ensure that gastric adenocarcinomas are displayed and adjusted in multiple directions to avoid missing details of different growth directions of different tumors. Resampling was not selected due to consistent layer thickness [26]. The characteristic data were not normalized because doing so could lead to the loss of some features [26]. The intergroup correlation coefficient and cross-validation were used to ensure that the radiomics feature selection of the tumor was repeatable.

Future research directions should include further external verification. A similar study could be conducted on colorectal cancer and other digestive tract cancers as well. The use of modalities other than CT, different sets of tested characteristics, or different algorithms could also be studied. Larger datasets could help to improve the generalization ability of our model. Our further investigation will focus on these research directions.

There are still some shortcomings in this study. First, the research is preliminary, with limited accumulation of data to date that could bring this to the clinic. Second, the size of the population sample was limited.

In conclusion, CT radiomics is of great value in predicting the expression of PD-L1 in patients with gastric adenocarcinoma. The diagnostic performance was improved by establishing a combined model of clinical factors and CT radiomics features.
